# Adiponectin to the rescue: extracorporeal photopheresis for managing immune checkpoint inhibitor-induced toxicities

**DOI:** 10.1038/s41392-025-02205-y

**Published:** 2025-04-11

**Authors:** Robin Reschke, Alexander H. Enk, Jessica C. Hassel

**Affiliations:** 1 Heidelberg University, Medical Faculty Heidelberg, Department of Dermatology and National Center for Tumor Diseases (NCT), NCT Heidelberg, a partnership between DKFZ and University Hospital Heidelberg, Heidelberg, Germany; 2 Germany German Cancer Consortium (DKTK), German Cancer Research Center (DKFZ) Core Center Heidelberg, Heidelberg, Germany

**Keywords:** Tumour immunology, Tumour immunology

In a recent study published in *Cancer Cell*, Braun et al. introduced extracorporeal photopheresis (ECP) as a novel immunomodulatory approach to mitigate immune-related adverse events (irAEs) associated with immune checkpoint inhibitors (ICIs) without compromising anti-tumor immunity.^1^ ECP suppressed Th1/Trm cell activation and neutrophil infiltration while enhancing an anti-inflammatory macrophage phenotype through adiponectin, facilitating the resolution of steroid-refractory irAEs.

Given the increasing application of ICIs in cancer therapy, ECP might offer a promising alternative to glucocorticoids and other immunosuppressive treatments that may diminish therapeutic efficacy.^2^ ECP is an immunomodulatory therapy initially developed for cutaneous T-cell lymphoma. Its clinical applications have expanded to include conditions such as acute graft-versus-host disease and bronchiolitis obliterans syndrome.^3^ The procedure involves leukapheresis, photoactivation, and reinfusion of treated cells. This article explores the mechanisms of action in immune regulation in the context of irAEs. Braun et al. investigated the impact of ECP vs. glucocorticoids on anti-PD-1 therapy in multiple cancer models.^1^ Their findings revealed that glucocorticoids treatment led to reduced survival, increased tumor burden, and diminished infiltration of tumor-specific T cells across various cancers, including melanoma, lung, colon, and renal cancers. Conversely, ECP preserved anti-PD-1 efficacy by maintaining tumor-specific T cell responses while also mitigating irAEs. The study also identified recipient-derived adiponectin as a key mediator of immunosuppressive effects through experiments using adiponectin-deficient mice. In an ICI-colitis mouse model adiponectin was most significantly upregulated in ECP-treated mice compared to controls. However, in these tumor-bearing mice, adiponectin expression in the tumor microenvironment (TME) remained unchanged between anti-PD-1 + ECP and anti-PD-1 monotherapy. In this dextran sodium sulfate + anti-PD-1 model, ECP prevented body weight loss and colon shortening. The positive effect of ECP was confirmed in additional models. Following ECP, lymphocyte and neutrophil recruitment to the colon was reduced in mice receiving T cells from ICI-colitis patients (humanized model). In a model with Rag2−/−Il2rg−/− mice, mice were injected with healthy donor PBMCs and anti-PD-1. ECP reduced histopathological markers of colitis. In a syngeneic mouse T cell transfer model, ECP therapy attenuated colitis exacerbation induced by anti-PD-1.

The role of adiponectin in resolving ICI-colitis was further confirmed through administration of the adiponectin receptor agonist “AdipoRon” or recombinant adiponectin, both of which synergized with ECP to reduce colonic inflammation. The study demonstrated that apoptotic leukocytes accumulating in inflamed tissues induced adiponectin. ECP-treated leukocytes experienced apoptosis, indicated by the stabilization of p53 and the cleavage of caspase-3/8/9 and poly (ADP-ribose) polymerase. In Rag2−/−Il2rg−/− mice, ECP led to the rapid clearance of transgenic splenocytes. To understand how ECP induces adiponectin expression, researchers co-cultured ECP-treated leukocytes with bone marrow-derived macrophages, which play a crucial role in regulating intestinal inflammation. The ECP-treated cells increased the expression of calreticulin, an “eat-me” signal that promoted their phagocytosis by the macrophages. SIINFEKL-loaded leukocytes were used to confirm phagocytosis and antigen cross-presentation.

Mechanistically, the uptake of ECP-treated splenocytes resulted in STAT6 phosphorylation and the upregulation of markers such as adiponectin, arginase-1 or CD206 in macrophages. These macrophages were found in the inflamed colon but not in tumors, suggesting preferential phagocytosis in colitis. Furthermore, myeloid cells showed high ARG1 expression hours after ECP, confirming the induction of an anti-inflammatory state. Conversely, blocking the phagocytosis of ECP-treated cells reduced STAT6 phosphorylation, M2 polarization, and adiponectin induction in bone marrow-derived macrophages. To characterize ECP-induced cell death, splenocytes subjected to freeze-thaw stress or camptothecin-induced apoptosis were analyzed. Although apoptosis levels were similar to those induced by ECP treatment, these alternative methods did not enhance calreticulin expression, phagocytosis, or M2 polarization. This indicates that only ECP-treated cells provide protection against ICI-colitis. Depleting colonic phagocytes with Clodrosomes in the dextran sodium sulfate + anti-PD-1 mouse model of colitis diminished ECP’s therapeutic efficacy, emphasizing the importance of phagocytosis in mediating its immunosuppressive effects.

A phase-1b/2 clinical trial provided further validation of ECP’s effectiveness. Among 14 patients with refractory irAEs, ECP achieved a 92% overall response rate, including a 100% complete remission rate for ICI-induced colitis. Patients exhibited a variety of irAEs, including colitis, hepatitis, arthritis, and dermatitis, suggesting ECP’s broad applicability in irAE management. Importantly, glucocorticoid use was reduced in all patients, emphasizing ECP’s role in limiting the need for systemic immunosuppression.

**Fig. 1:**
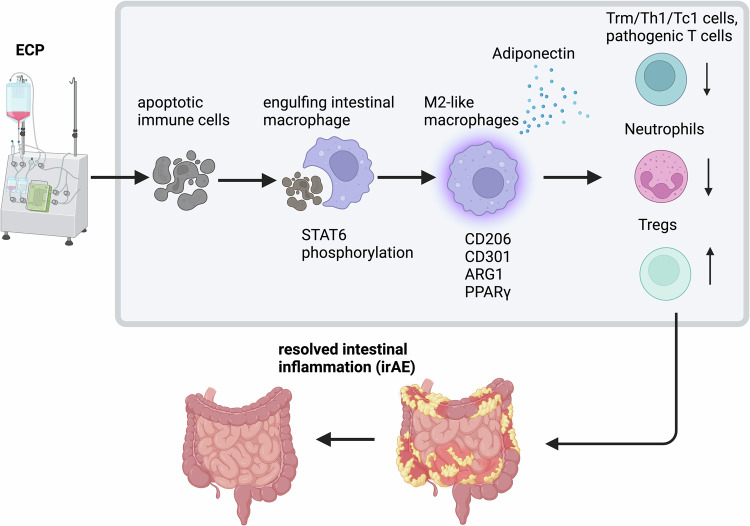
Schematic representation of ECP’s effect on immune cells and intestinal irAEs. ECP induces leukocyte apoptosis, followed by their phagocytosis by macrophages. Signal transducer and activator of transcription 6 (STAT6) is phosphorylated and induces Arginase-1 (ARG1), CD206, CD301 and peroxisome proliferator-activated receptor gamma (PPARγ) in macrophages. These macrophages adopt an M2-like phenotype produce adiponectin. Consequently, tissue-resident memory T cells (TRMs), Th1, Tc1, pathogenic CD25^high^ T cells and neutrophils are downregulated. Conversely, regulatory T cells (Tregs) are downregulated. The immunological changes lead to the resolution of irAE-associated inflammation in the gastrointestinal tract. ECP: extracorporeal photopheresis. (figure created with BioRender.com)

Prior studies have shown that tissue-resident memory T (Trm) cells, particularly Tc1 and Th1 polarized subsets, play a role in mediating cutaneous and gastrointestinal irAEs.^4^ Braun et al. corroborated these findings, demonstrating increased activation and proliferation of CD8+ Trm cells with elevated IFN-γ production in irAEs.^1^ ECP therapy significantly reduced Trm cell populations, underscoring their involvement in irAEs. Additionally, pathogenic T cells, Th1/Tc1 cells, and neutrophils decreased following ECP treatment. However, the frequency of regulatory T cells increased (Fig. 1).

While this study provides promising insights, the small patient cohort highlights the need for larger trials with longer follow-up data to comprehensively assess ECP’s effects on immunotherapy outcomes and immune markers. Notably, Tc1/Th1 and Trm cells are critical for both irAE pathogenesis and anti-tumor responses.^2,4^ If their suppression via the adiponectin–M2 macrophage axis affects tumor immunity, it could influence treatment strategies. Furthermore, evidence from breast cancer patients suggests that adiponectin plays a role in priming T cells through dendritic cells (DCs),^5^ which express high levels of adiponectin receptors (AdipoR1 and AdipoR2). These receptors modulate immune responses via distinct pathways—AdipoR1 enhances IL-10 production through AMPK and MAPKp38 activation, while AdipoR2 regulates inflammation via COX-2 and PPARγ signaling. Together, these pathways inhibit NF-κB in DCs and reduces their capability to induce antigen-specific T cell responses. The long-term effect of ECP on the TME and anti-tumor immunity in patients could be monitored with sequential biopsies and peripheral immune cell monitoring [2]. In addition, distinct ICIs might induce different immunological changes and associated toxicities. Future large-scale trials with longer follow-up will be crucial to validate these findings and establish ECP’s efficacy in managing irAEs across diverse ICI regimens (anti-PD-1/PD-L1, anti-CTLA-4, anti-LAG-3). A larger German clinical trial (NCT05700565) is currently enrolling patients to evaluate ECP’s efficacy in steroid-refractory cases. Overall, ECP emerges as a promising treatment option for refractory irAEs, with the potential to decouple irAE management from ICI efficacy. However, its superiority over other strategies, such as Interleukin-6 receptor blockade (e.g. tocilizumab), remains to be determined. Additionally, evaluating ECP’s effectiveness in long-term or late-onset ICI-induced toxicities warrants further investigation.
